# Substrate recognition mode of a glycoside hydrolase family 42 β-galactosidase from *Bifidobacterium longum* subspecies *infantis* (*Bi*Bga42A) revealed by crystallographic and mutational analyses

**DOI:** 10.20517/mrr.2023.14

**Published:** 2023-05-26

**Authors:** Aina Gotoh, Masafumi Hidaka, Haruko Sakurama, Mamoru Nishimoto, Motomitsu Kitaoka, Mikiyasu Sakanaka, Shinya Fushinobu, Takane Katayama

**Affiliations:** ^1^Graduate School of Biostudies, Kyoto University, Sakyo-ku, Kyoto 606-8502, Japan.; ^2^Ishikawa Prefectural University, Nonoichi, Ishikawa 921-8836, Japan.; ^3^Graduate School of Agricultural Science, Tohoku University, Sendai, Miyagi 980-8572, Japan.; ^4^Institute of Food Research, National Agriculture and Food Research Organization, Tsukuba, Ibaraki 305-8642, Japan.; ^5^Faculty of Agriculture, Niigata University, Niigata 950-2102, Japan.; ^6^Department of Biotechnology, The University of Tokyo, Bunkyo-ku, Tokyo 113-8657, Japan.

**Keywords:** lacto-*N*-tetraose, glycoside hydrolase family 42, β-galactosidase, bifidobacteria, human milk oligosaccharides, crystal structure

## Abstract

**Aim:** *Bifidobacterium longum* subsp. *infantis* uses a glycoside hydrolase (GH) family 42 β-galactosidase (*Bi*Bga42A) for hydrolyzing lacto-*N*-tetraose (LNT), which is the most abundant core structure of human milk oligosaccharides (HMOs). As such, *Bi*Bga42A represents one of the pivotal enzymes underpinning the symbiosis between bifidobacteria and breastfed infants. Despite its importance, the structural basis underlying LNT hydrolysis by *Bi*Bga42A is not understood. Moreover, no substrate-complexed structures are available to date for GH42 family members.

**Methods:** X-ray crystallography was used to determine the structures of *Bi*Bga42A in the apo- and liganded forms. The roles of the amino acid residues that were presumed to be involved in catalysis and substrate recognition were examined by a mutational study, in which kinetic parameters of each mutant were determined using 4-nitrophenyl-β-D-galactoside, lacto-*N*-biose I, LNT, and lacto-*N*-neotetraose (LNnT) as substrates. Conservation of those amino acid residues was examined among structure-determined GH42 β-galactosidases.

**Results:** Crystal structures of the wild-type enzyme complexed with glycerol, the E160A/E318A double mutant complexed with galactose (Gal), and the E318S mutant complexed with LNT were determined at 1.7, 1.9, and 2.2 Å resolutions, respectively. The LNT molecule (excluding the Gal moiety at subsite +2) bound to the E318S mutant is recognized by an extensive hydrogen bond network and several hydrophobic interactions. The non-reducing end Gal moiety of LNT adopts a slightly distorted conformation and does not overlap well with the Gal molecule bound to the E160A/E318A mutant. Twelve of the sixteen amino acid residues responsible for LNT recognition and catalysis in *Bi*Bga42A are conserved among all homologs including β-1,6-1,3-galactosidase (*Bl*Gal42A) from *Bifidobacterium animalis* subsp. *lactis*.

**Conclusion:** *Bl*Gal42A is active on 3-β-galactobiose similarly to *Bi*Bga42A but is inactive on LNT. Interestingly, we found that the entrance of the catalytic pocket of *Bl*Gal42A is narrower than that of *Bi*Bga42A and seems not easily accessible from the solvent side due to the presence of two bulky amino acid side chains. The specificity difference may reflect the structural difference between the two enzymes.

## INTRODUCTION

Human milk not only fulfills the nutritional requirements of newborns but also guides microbiota development in the infant gut^[1]^ Human milk oligosaccharides (HMOs), a collective term for sugars with a degree of polymerization of ≥ 3, are bioactive compounds shown to be instrumental in the formation of bifidobacteria-rich gut microbiota^[2-6]^. HMOs are present at a concentration of 10~20 g/L in breast milk and are the third most abundant solid material after lactose (Lac) and lipids. HMOs are assumed to reach the colon intact because they are resistant to pancreatic digestion^[7]^. A unique feature of HMOs that distinguishes them from other mammalian milk oligosaccharides is the richness of type-1 chains (Galβ1-3GlcNAc-*O*-R)^[8]^. As such, lacto-*N*-tetraose (LNT, Galβ1-3GlcNAcβ1-3Galβ1-4Glc) is present as the most abundant core structure of HMOs^[3,8,9]^. The type-1 chain is resistant to most bacterial β-galactosidases^[3]^, and its hydrolysis requires a specific subgroup of glycoside hydrolase family (GH) 42 enzymes that infant gut-associated bifidobacterial species possess. We previously reported that *Bifidobacterium longum* subsp. *infantis* (*B. infantis*) ATCC 15697, which directly internalizes HMOs into its cells using ABC transporters^[3,10]^, encodes three intracellular GH42 homologs in its genome. Among the three paralogs, only a β-galactosidase termed *Bi*Bga42A (Blon_2016) hydrolyzes LNT^[11]^. One of the remaining two GH42 enzymes, *Bi*Bga42B (Blon_2013), was later shown to be specific for β-1,4-galactooligosaccharides^[12]^. A study by James *et al*. showed that inactivation of *lntA*, a *Bi*Bga42A homolog gene (Bbr_0529), in *Bifidobacterium breve* UCC2003 abolishes the ability of the strain to grow on LNT as a sole carbon source, while the mutation neither affects its ability to utilize Lac nor lacto-*N*-neotetraose (LNnT, Galβ1-4GlcNAcβ1-3Galβ1-4Glc)^[13]^. The genome of *B. breve* UCC2003 encodes an additional GH42 homolog^[14]^, although its specificity has not been determined. GH42 members have thus acquired diversified functions during evolution. Note that *B. bifidum* and several strains of *B. longum* subsp. *longum* (*B. longum*) employ an alternative strategy to degrade LNT. These bifidobacteria extracellularly degrade LNT into lacto-*N*-biose I (LNB) and lactose (Lac) by lacto-*N*-biosidases belonging to GH20^[15,16]^ (for *B. bifidum*) and GH136^[5,17,18]^ (several strains of *B. longum*). The released LNB is then imported by a specific ABC transporter (GltABC) for assimilation^[19,20]^.

As our diet contains different sets of β-galactosides, which are represented by HMOs during breastfeeding and by galactan oligosaccharides after weaning, precise characterization of GH42 members is of fundamental importance to obtain a better understanding of how the gut microbiota is shaped and affected by dietary β-galactosides during the early stages of life.

Since our first report of the crystal structure of a GH42 β-galactosidase from *Thermus thermophilus* A4^[23]^, the three-dimensional structures of 8 members of GH42 have been reported^[24-31]^. Nonetheless, only a few GH42 members have been subjected to substrate specificity analysis, and crystal structures of GH42 enzymes are available only in the apo form and/or the product Gal-complexed form. Here, we report the crystal structures of *Bi*Bga42A wild-type (WT) enzyme complexed with glycerol, E160A/E318A mutant complexed with Gal, and E318S mutant complexed with LNT. This study is the first to show a substrate-complexed form of GH42 members.

## METHODS

### Chemicals

4-Nitrophenyl-β-D-galactoside (*p*NP-Gal) was purchased from Wako Pure Chemical Industries (Osaka, Japan). LNT and LNnT were provided as gifts from Glycom A/S (Hørsholm, Denmark). LNT was further purified by the method described by Ojima *et al*.^[32]^. LNB was prepared as described previously^[33]^. Other reagents of analytical grade were obtained from commercial sources.

### Site-directed mutagenesis

The QuikChange site-directed mutagenesis method (Agilent Technologies, CA, USA) was used for introducing amino acid substitutions. pET23b (Merck Millipore, MA, USA) carrying the gene for WT *Bi*Bga42A with a C-terminal hexahistidine-tag [Bga42A-(His)_6_] was used as the template^[11]^. The following primers and their complementary strands were used for the mutagenesis (mutation sites are underlined): 5'-gccagcccggtgccgcacagcactggcgcgcc-3' (for R121A), 5'-catgtgagcaacgcgtacggctgccac-3' (for E160A), 5'-cgacggcaacgcaatgaacccggg-3' (for F221A), 5'-cagaccaccaacttcggcgtctccgcg-3' (for M262G), 5'- tccaacgaccatttcttctcgcccggc-3 (for Y287F), 5'-tggttcctcatggcgcattccacgtcc-3' (for E318A), 5'-tggttcctcatgggccattccacgtcc-3' (for E318G), 5'-tggttcctcatgcagcattccacgtcc-3' (for E318Q), 5'-tggttcctcatgagccattccacgtcc-3' (for E318S), 5'-ccgccgtcaacgcacgcccgaccaac-3' (for W326A), and 5'-cgtcaactgggcaccgaccaactac-3' (for R327A). After sequence confirmation, respective plasmids were introduced into the *Escherichia coli* BL21 (DE3) Δ*lacZ* strain for expression^[11]^.

### Expression and purification of Bga42A variants

The WT Bga42A enzyme from *B. infantis* ATCC 15697 and its mutant enzymes were expressed and purified by a previously described method^[12]^, which involved Ni-nitrilotriacetic acid (NTA) affinity chromatography (Qiagen, Hilden, Germany), MonoQ 5/50 GL anion exchange chromatography (GE Healthcare Bio-Sciences, Uppsala, Sweden), and Superdex 200 10/300 GL size exclusion chromatography (GE Healthcare Bio-Sciences). When comparing LNT-hydrolyzing activity among E318A, E318G, E318Q, and E318S mutants, the enzymes were purified by Ni-NTA column chromatography only. The purified enzymes were concentrated using Amicon Ultra Centrifugal Filters (30 K) (Merck Millipore), dialyzed against 50 mM sodium phosphate buffer (pH 7.0) containing 0.05% Tween-20, and stored at 4 ºC. The enzymes were stable for at least one month under the conditions. The protein was quantified using a theoretical absorption coefficient at 280 nm, calculated based on the sequence (https://web.expasy.org/protparam/).

### Enzyme assay

*p*NP-Gal, LNB, LNT, and LNnT were used as the substrates. The release of Gal was colorimetrically quantified as described previously^[12,34]^, in which the released Gal was continuously converted to 6-phospho-glucono-1,5-lactone with the production of an equimolar of Thio-NADH. The reaction mixture contained varied concentrations of each of the substrates in 100 mM citrate-phosphate buffer (pH 6.5) supplemented with 10 U/mL hexose mutarotase (FUJIFILM Wako Pure Chemical, Osaka, Japan), 5 U/mL galactokinase, 2.5 U/mL UDP-glucose-hexose-1-phosphate uridylyltransferase, 10 U/mL phosphoglucomutase (Sigma-Aldrich, MO, USA), 5 U/mL glucose 6-phosphate dehydrogenase (Sigma-Aldrich), 1 mM UDP-Glc, 1 mM ATP, 0.625 mM Thio-NAD^+^ (Oriental Yeast, Tokyo, Japan), 0.0125 mM Glc-1,6-bisphosphate, and 12.5 mM MgCl_2_ in a total volume of 40 μL. Galactokinase (GalK, BLLJ_0339) and UDP-glucose-hexose-1-phosphate uridylyltransferase (GalT, BLLJ_0398) from *B*. *longum* JCM 1217 were prepared as described previously^[33,34]^. The mixture was preincubated at 37 ºC for 5 min, to which 10 μL of similarly preincubated enzyme solution diluted with 50 mM sodium phosphate buffer (pH 6.5) containing 0.05% Tween-20 was added to initiate the reaction. The reaction was monitored at 37 ºC by measuring the absorbance at 400 nm (Thio-NADH) every 2 min for 120 min. A Multiskan GO microplate reader (Thermo Fisher Scientific, MA, USA) was used for spectrophotometry. The kinetic parameters were calculated by curve-fitting the experimental data with the Michaelis-Menten equation, using KaleidaGraph 4.0 (Synergy Software, Tokyo, Japan).

### Crystallography

The WT enzyme, E160A/E318A double mutant, and E318S mutant were purified as described above. The WT and E160A/E318A proteins were dialyzed against 20 mM 2-[4-(2-hydroxyethyl)piperazin-1-yl]ethanesulfonic acid-KOH buffer (pH 7.0) containing 150 mM NaCl, while E318S protein was dialyzed against 20 mM 3-(*N*-morpholino)propanesulfonic acid (MES)-NaOH buffer (pH 6.0) containing 0.05% Tween-20. The hanging drop vapor diffusion method was used for crystallization. The crystal of WT complexed with glycerol was obtained by mixing 1 μL of a protein solution (20 mg/mL) containing 100 mM Gal with an equal volume of a reservoir solution consisting of 0.1 M KSCN, 30% PEG MME 2000, and 25% glycerol (cryoprotectant). The crystal of E160A/E318A complexed with Gal was obtained by mixing 1 μL of a protein solution (20 mg/mL) containing 60 mM LNT with an equal volume of a reservoir solution consisting of 0.1 M MES-NaOH buffer (pH 6.5), 0.1 M (CH_3_COO)_2_Mg, 10% PEG 10000, and 25% glycerol (cryoprotectant). The crystal of E318S complexed with LNT was obtained by mixing 1 μL of a protein solution (20 mg/mL) containing 100 mM LNT with an equal volume of a reservoir solution consisting of 5 mM MES-NaOH buffer (pH 5.8), 0.1 M KSCN, 30% PEG MME 2000, and 20% ethylene glycol (cryoprotectant). The crystals grew at 20 ºC within 2 days in all cases. The crystals were flash-cooled in a nitrogen stream at 100 K. X-ray diffraction data were collected at 100 K at the beamline BL-5A at the Photon Factory of the High Energy Accelerator Research Organization (KEK, Tsukuba, Japan, λ = 1.0 Å). Preliminary diffraction data were collected at other beamlines at Photon Factory and SPring-8 (Hyogo, Japan). The data sets were processed using XDS (Jan 10, 2022)^[35]^ and Aimless (0.7.9)^[36]^. Molecular replacement was performed using MOLREP (11.9.02)^[37]^. Model building and refinement were performed using Coot (0.9.8.6)^[38]^ and Refmac (5.8.0403)^[39]^. The Dali Server was used for structural comparison^[40]^. Molecular interface analysis was performed using the PDBePISA server^[41]^. Molecular graphic images were prepared using PyMOL (2.5.4) (Schrödinger, NY, USA). Stereographic figures were created using the “ray angle =” command, specifying -3 and +3 degrees for the left and right panels, respectively.

### Phylogenetic tree construction

The structure-determined β-galactosidases^[23-30]^ and α-arabinopyranosidase^[31]^ listed in GH42 of the Carbohydrate-Active enZYmes database^[42]^ and *Bi*Bga42A were used for the tree construction. A β-galactosidase from *Bifidobacterium adolescentis* was omitted from the analysis because the structure does not contain the catalytic domain (Midwest Center for Structural Genomics). The maximum likelihood tree was constructed using MegaX, based on the sequences aligned using the ClustalW algorithm with default settings^[43]^.

### Search for *Bi*Bga42A homologs among several bifidobacterial species

The occurrence of *Bi*Bga42A homologs among several *Bifidobacterium* species was examined using the Tblastn program (https://blast.ncbi.nlm.nih.gov/Blast.cgi). Genome sequence-completed, type strains of *B. adolescentis* ATCC 15703, *Bifidobacterium angulatum* JCM 7096, *Bifidobacterium animalis* subsp. *animalis* ATCC 25527, *B. animalis* subsp. *lactis* (*B. lactis*) DSM 10140, *B. bifidum* JCM 1255, *B. breve* JCM 1192, *Bifidobacterium catenulatum* subsp. *catenulatum* JCM 1194, *B. catenulatum* subsp. *kashiwanohense* JCM 15439, *Bifidobacterium dentium* JCM 1195, *Bifidobacterium eulemuris* DSM 100216, *Bifidobacterium lemurum* DSM 28807, *B. longum* JCM 1217, *Bifidobacterium pseudocatenulatum* JCM 1200, and *Bifidobacterium pseudolongum* subsp. *globosum* DSM 20092 were used for the homolog search. Alignment of the retrieved homologs was carried out using the ClustalW program with default settings^[44]^.

## RESULTS AND DISCUSSION

### Crystal structures of *Bi*Bga42A

**Figure 1:**
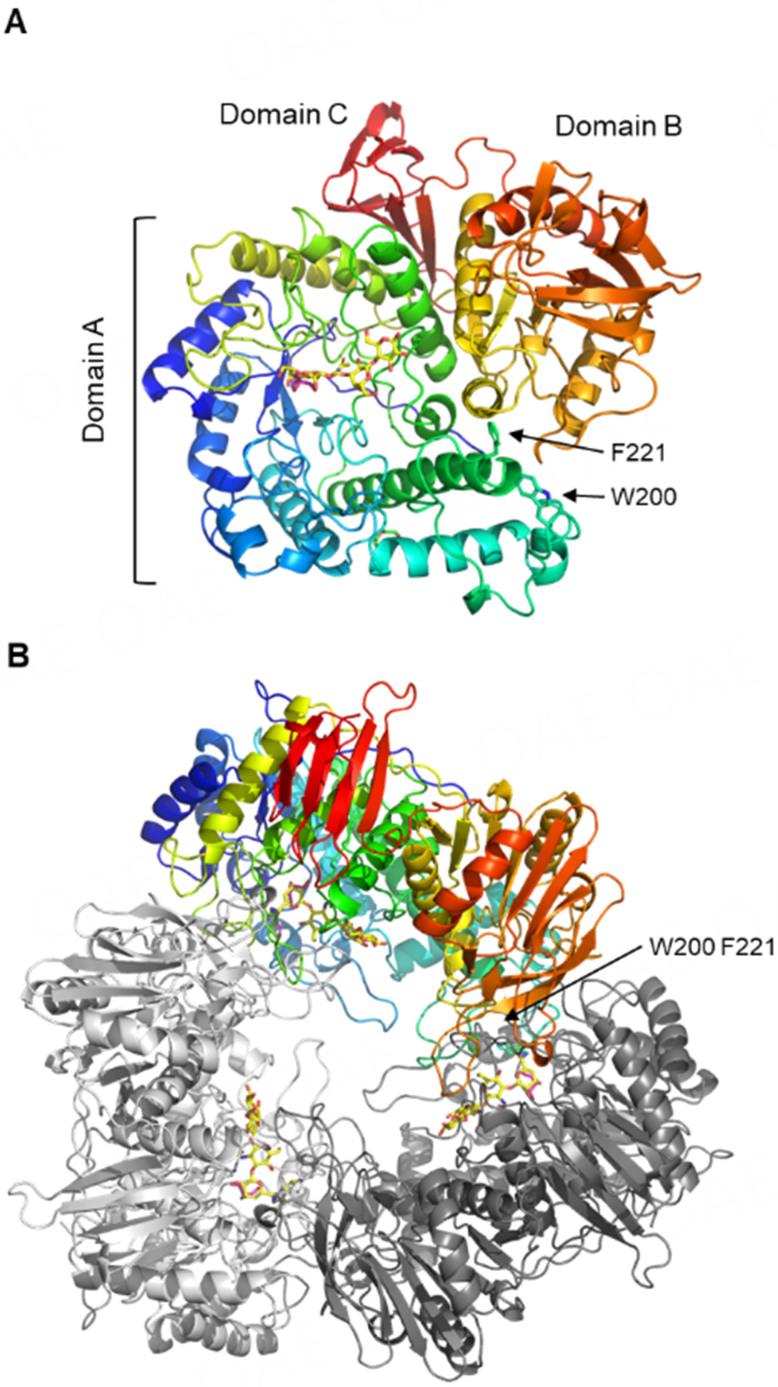
The overall structure of *Bi*Bga42A. WT-GOL structure (rainbow color) with bound glycerol (magenta) is shown. LNT in E318S-LNT structure (yellow) is superimposed [Figure 2]. (A) Monomer structure; (B) Trimer structure related by a crystallographic 3-fold axis. Trp-200 and Phe-221, which form the substrate binding site of a neighboring subunit, are indicated by arrows. *Bi*Bga42A: a glycoside hydrolase family 42 β-galactosidase; LNT: lacto-*N*-tetraose; WT-GOL: WT enzyme complexed with glycerol.

First, we determined the structure of the WT enzyme complexed with glycerol (WT-GOL) at 1.7 Å resolution [Supplementary Table 1]. The electron density of a glycerol molecule, which was used as a cryoprotectant, was observed at the active site, even though the crystals were grown in the presence of Gal [Supplementary Figure 1A]. *Bi*Bga42A has a three-dimensional structure similar to other GH42 enzymes that have three domains consisting of a (β/α)_8_ barrel (domain A), an α/β fold (domain B), and an anti-parallel β-sandwich (domain C) [Figure 1A]. A Dali structural search^[45]^ revealed that *Bi*Bga42A is most similar to GH42 β-1,6-galactosidase from *B. bifidum* S17 (BbgII, PDB ID: 4UCF)^[24]^ with the root-mean-square deviation (RMSD) = 0.6 Å for 684 Cα atoms (Z score = 56.6 and sequence identity = 76%). The second hit was GH42 β-1,6-1,3-galactosidase from *B. lactis* Bl-04 (*Bl*Gal42A, PDB ID: 4UOZ)^[25]^ with RMSD = 0.6 Å for 685 Cα atoms (Z score = 55.9 and sequence identity = 62%). The asymmetric unit of the WT-GOL crystal contained one molecule of *Bi*Bga42A, and it forms a homotrimer with symmetry-related molecules by a crystallographic 3-fold axis [Figure 1B]. A PISA molecular interface analysis calculated that approximately 27% of the entire surface area of the trimer (17,082 Å of 64,278 Å) was buried. Our previous study using calibrated gel filtration chromatography also suggested that *Bi*Bga42A forms a trimer in solution^[11]^. The trimeric structure of *Bi*Bga42A resembles a flowerpot, like other GH42 enzymes^[23-25]^.

**Figure 2:**
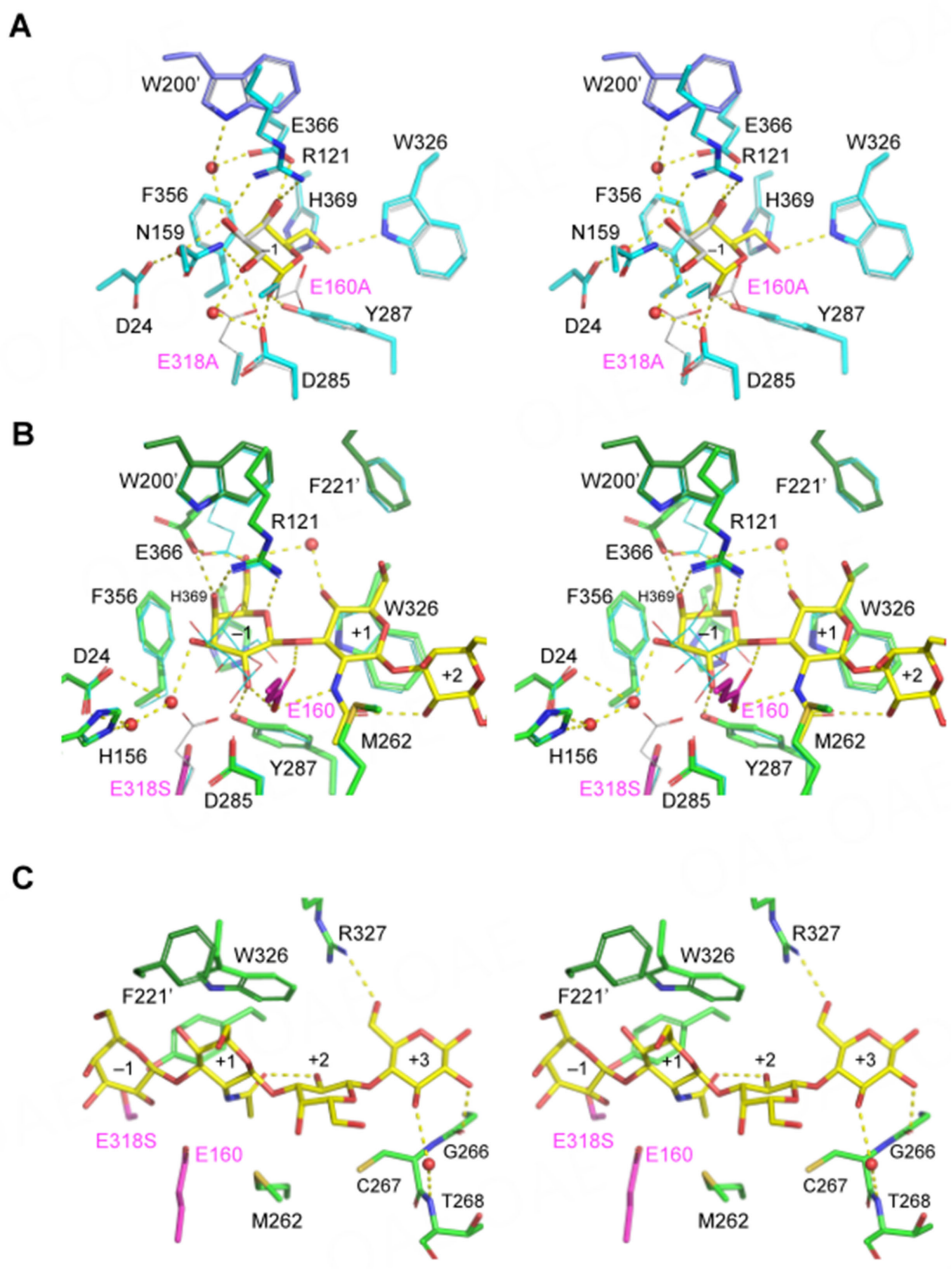
Stereoviews of the active site of *Bi*Bga42A. (A) E160A/E318A-Gal (cyan) and bound α-Gal (yellow) is superimposed with WT-GOL (white, thin sticks except for glycerol). W200 and F221 are from the neighboring molecule (light purple); (B) and (C) E318S-LNT (green) and bound LNT (yellow) focused on LNB (Galβ1-3GlcNAc) disaccharide structure bound in subsites -1 and +1 (B) and Lac (Galβ1-4Glc) disaccharide structure in subsites +2 and +3 (C). In (B), E160A/E318A-Gal (cyan) and the side chain of Glu-318 in WT-GOL (white) are superimposed as thin sticks. W200 and F221 are from the neighboring molecule (dark green). *Bi*Bga42A: a glycoside hydrolase family 42 β-galactosidase; LNT: lacto-*N*-tetraose; WT-GOL: WT enzyme complexed with glycerol.

Glu-160 and Glu-318 in *Bi*Bga42A were predicted to be the acid/base catalyst and the nucleophile, respectively, based on previous studies of GH42 enzymes and sequence alignment^[46,47]^. The *k*_cat_ values of the respective alanine mutants (E160A and E318A) towards *p*NP-Gal dropped by 170- and 2,900-fold compared to the WT enzyme, respectively, without affecting *K*_m_ values (within 2.5-fold change) [Table 1 and Supplementary Figure 2]. Based on these results, we first attempted co-crystallization of E318A with LNT. However, in a preliminary experiment, we found that the purified mutant can hydrolyze LNT when present at a high concentration (9 mg/mL) [Supplementary Figure 2]. Therefore, we also replaced the acid/base catalyst Glu-160 with alanine and performed co-crystallization with LNT using the preparation (E160A/E318A double mutant). The crystals obtained within 2 days were used for X-ray diffraction data collection. However, LNT was also hydrolyzed during the crystallization under these conditions, because only the electron density of the product Gal in the α-configuration (α-Gal) was observed in subsite -1 of the active site [Supplementary Figure 1B]. The Gal-complexed structure was determined at 1.9 Å resolution (E160A/E318A-Gal) [Supplementary Table 1]. α-Gal was also observed in the crystal structures of several other GH42 enzymes^[23-26]^. The asymmetric unit contained six molecules (two trimers) of *Bi*Bga42A. The main chain structures of the six polypeptides (chains A-F) were virtually the same (Cα RMSD < 0.107 Å for all chain pairs), and an α-Gal molecule was similarly bound in each chain. Therefore, we mainly describe the chain A molecule. The pyranose ring of the α-Gal adopts an undistorted ^4^*C*_1_ conformation, and the C6 hydroxyethyl group takes a *gt* conformation [Figure 2A and Supplementary Figure 1B]. The glycerol molecule in WT-GOL occupies the C2-C4 position of the Gal. The Gal recognition by the protein involves

**Table 1:** The kinetic parameters of *Bi*Bga42A variants for *p*NP-Gal, LNB, LNT, and LNnT

		***p*NP-Gal**	**LNB**	**LNT**	**LNnT**
**WT**	*K*_m_ (mM)	0.57	25	2.7	15
	*k*_cat_ (s^-1^)	580	31	86	15
					
**R121A**	*K*_m_ (mM)	1.4	42	16	35
	*k*_cat_ (s^-1^)	32	0.79	3.6	0.53
					
**E160A**	*K*_m_ (mM)	0.94	nd*^b^*	nd	nd
	*k*_cat_ (s^-1^)	3.4	nd	nd	nd
					
**F221A*^a^***	*K*_m_ (mM)	1.2	27	8.4	24
	*k*_cat_ (s^-1^)	110	1.4	11	6.4
					
**M262G**	*K*_m_ (mM)	1.1	14	3.0	13
	*k*_cat_ (s^-1^)	12	0.99	2.9	0.13
					
**Y287F**	*K*_m_ (mM)	1.0	16	2.6	18
	*k*_cat_ (s^-1^)	6.0	0.18	1.1	0.13
					
**E318A**	*K*_m_ (mM)	1.4	nd	nd	nd
	*k*_cat_ (s^-1^)	0.20	nd	nd	nd
					
**W326A**	*K*_m_ (mM)	12	33	7.7	64
	*k*_cat_ (s^-1^)	44	0.054	0.14	0.13
					
**R327A**	*K*_m_ (mM)	0.92	53	11	52
	*k*_cat_ (s^-1^)	380	21	60	11
					

^a^The amino acid residue from the neighboring subunit in the trimer; *^b^*not determined. *Bi*Bga42A: a glycoside hydrolase family 42 β-galactosidase; LNB: lacto-*N*-biose I; LNT: lacto-*N*-tetraose; LNnT: lacto-*N*-neotetraose; *p*NP-Gal: 4-Nitrophenyl-β-D-galactoside; WT: wild-type.

**Figure 3:**
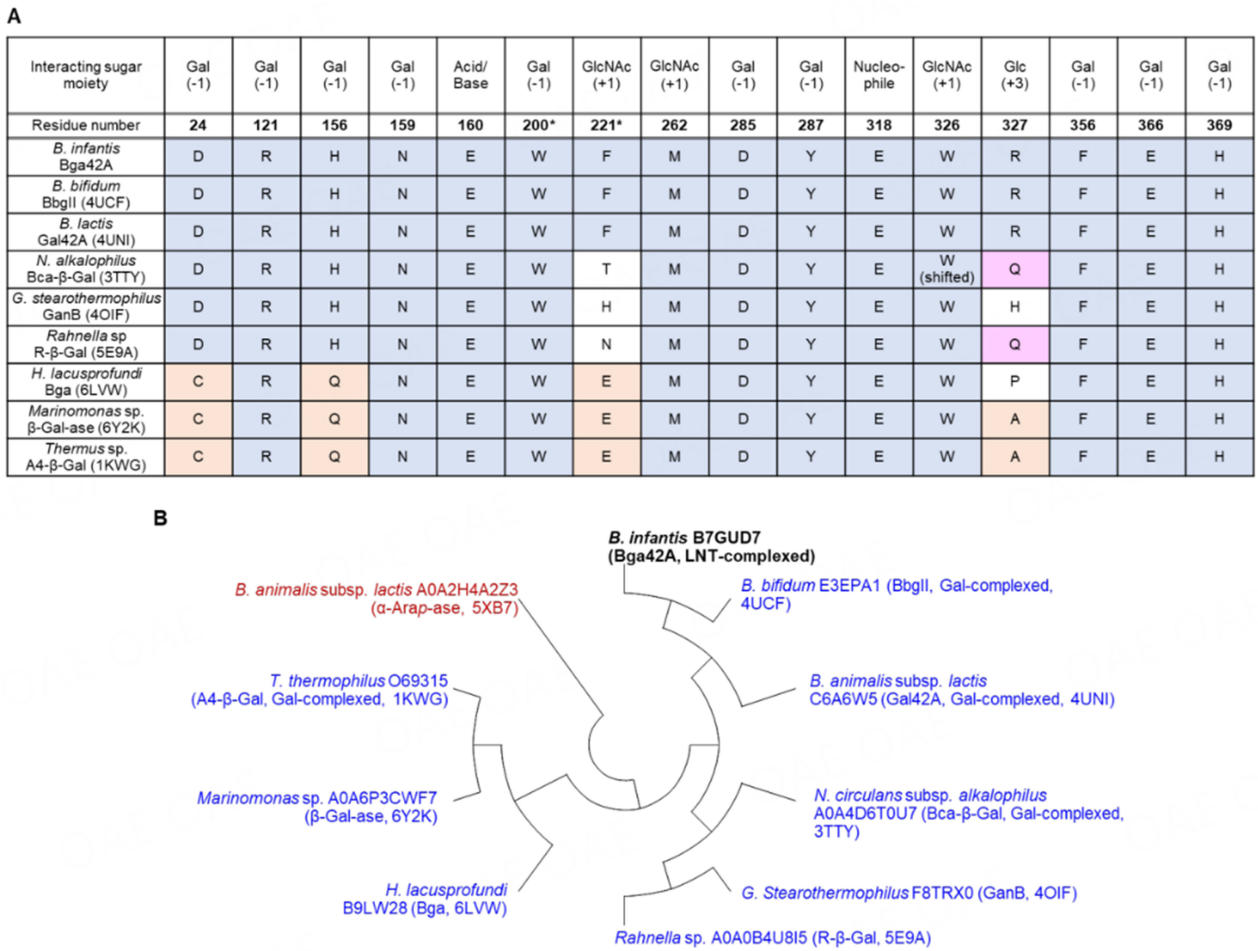
Sequence comparison within the structure-determined GH42 members. (A) The amino acid residues involved in ligand recognition in *Bi*Bga42A were aligned with eight structure-determined GH42 β-galactosidases using the ClustalW program^[44]^. The residue numbers are based on *Bi*Bga42A. The sugar moieties interacting with the respective amino acid residues are indicated by their subsite positions. Asterisks indicate the residues from the neighboring subunit; (B) Structure-determined enzymes, i.e., eight β-galactosidases (blue) and one α-arabinopyranosidase (brown), and *Bi*Bga42A (bold black) were used for the tree construction. The Uniprot numbers, PDB IDs, and taxonomic names are shown. β-Galactosidase from *B. adolescentis* (PDB ID: 5VYM) was omitted from the analysis because the structure does not contain the catalytic domain. The maximum likelihood tree was constructed using MegaX based on the sequences aligned using ClustalW with default settings^[43]^. The lowest amino acid identity detected among the members is 20% between *B. lactis* A0A2H4A2Z3 and *B. bifidum* E3EPA1, *G. stearothermophilus* F8TRX0, or *Thermus* sp. O69315, while the highest one is 76% between *B. infantis* B7GUD7 (*Bi*Bga42A) and *B. bifidum* E3EPA1 (BbgII). The sequences are: *B. bifidum* E3EPA1^[24]^, *B. animalis* subsp. *lactis* C6A6W5^[25]^, *N. circulans* subsp. *alkalophilus* A0A4D6T0U7^[26]^, *G. stearothermophilus* F8TRX0^[27]^, *Rahnella* sp. A0A0B4U8I5^[28]^, *H. lacusprofundi* B9LW28^[29]^, *Marinomonas* sp. A0A6P3CWF7^[30]^, *T. thermophilus* O69315^[23]^, and *B. animalis* subsp. *lactis* A0A2H4A2Z3^[31]^. *Bi*Bga42A: a glycoside hydrolase family 42 β-galactosidase; GH: glycoside hydrolase.

many hydrogen bonds and a stacking interaction. The O6 atom of the sugar forms hydrogen bonds with the side chain nitrogen atoms of Trp-326 and His-369, while the axial O4 atom is recognized by Arg-121 and Glu-366. Arg-121 is also involved in the recognition of the O3 atom of the sugar. The O3 atom also forms water-mediated hydrogen bonds with the Nε1 atom of Trp-200 from the neighboring subunit of the trimer. Trp-200 is located at the tip of a long helix in domain A [Figure 1]. The O2 atom of the Gal makes hydrogen bonds with the side chains of Asn-159 and Asp-285. The O2 atom and Asp-285 also form a hydrogen bond via a water molecule. The water molecule is located at the corresponding position of a side chain oxygen atom of Glu-318 (nucleophile) observed in WT-GOL. The α-anomeric O1 atom is recognized by the side chains of Asp-285 and Tyr-287 via hydrogen bonds. The side chain of Phe-356 makes a stacking interaction with the hydrophobic C4 region of the pyranose ring. All of these residues are conserved within the members of GH42 β-galactosidases with reported structures [Figure 3A]. In the crystal structure of the β-galactosidase from *Niallia circulans* (formerly called *Bacillus circulans*) subsp. *alkalophilus* (Bca-β-Gal), the residue corresponding to Trp-326 of *Bi*Bga42A is shifted away^[26]^.

As mentioned above, even in the E160A/E318A double mutant, the substrate hydrolysis occurred within 2 days of crystal growth. Crystal soaking experiments were unsuccessful. We then replaced Glu-318 with glycine (E318G), glutamine (E318Q), or serine (E318S). Using the His-tag affinity-purified preparations at high concentrations (9 mg/ml), we examined their remaining LNT-hydrolyzing activity. As a result, the lowest activity was detected for the E318S mutant [Supplementary Figure 2]. An LNT-complexed crystal was indeed obtained for the mutant, and the structure was determined at 2.2 Å resolution (E318S-LNT) [Supplementary Table 1]. The asymmetric unit of the E318S-LNT crystal contained two molecules (chains A and B) of *Bi*Bga42A, and they are virtually the same (Cα RMSD = 0.106 Å). Each chain in the asymmetric unit forms a trimer with crystallographic symmetry-related molecules. The electron density map of LNT was clearly visible at the active site [Supplementary Figure 1C]. We mainly describe chain B because the electron density of LNT was more clearly observed. The pyranose sugar ring of the Gal moiety in subsite -1 adopts a slightly distorted conformation toward ^O^*H*_5_, and the C6 hydroxymethyl group takes a *gg* rotamer conformation. The Cremer-Pople parameters (*ψ*, *φ*, and *Q*)^[48]^ of the Gal in subsite -1 are 324.6°, 29.3°, 0.559 and 323.6°, 28.6°, 0.566 for chains A and B, respectively. The O6 atom of the Gal moiety makes a water-mediated intramolecular hydrogen bond with the O4 atom of the GlcNAc moiety within the LNT molecule [Figure 2B]. The conformational change at the O6 atom of the Gal moiety resulted in the loss of two hydrogen bonds formed with Trp-326 and His-369 in the E160A/318A-Gal [Figure 2A]. Loss of hydrogen bonds is also found between the O3 atom of the Gal moiety and the side chain of Arg-121 and between the O2 atom of the Gal moiety and the side chains of Asn-159 and Asp-285. In E318S-LNT, the O3 atom of the Gal interacts with the side chains of Asp-24 and His-156 via water-meditated hydrogen bonds. The O2 atom of the Gal in E318S-LNT forms hydrogen bonds with the side chains of Glu-160 and Tyr-287, the latter interacting with the anomeric O1 atom of the α-Gal in E160A/E318A-Gal [Figure 2A]. The carboxyl group of Glu-160 is suitably poised for proton donation to the leaving group as it is located 3.3 Å from the glycosidic bond oxygen of the LNB (Galβ1-3GlcNAc) unit in LNT. The stacking interaction between the phenolic ring of Phe-356 and the C4 region of the Gal remains unchanged. As the Gal-recognition modes between E160A/E318A-Gal and E318S-LNT differ, the two Gal do not overlap well with each other even at the C1 atom [Figure 2B], to which the nucleophile Glu-318 residue is thought to attack to form a galactosyl-enzyme intermediate during catalysis^[47]^. The C1 atom of the Gal moiety at subsite -1 in E318S-LNT has moved away from the side chain of Ser-318 with a distance of 6.2 Å. In addition, the C1 atom was located 4.3 Å from the nearest side chain oxygen atom (Oε1) of the superimposed Glu-318 of WT-GOL, and the configuration was not suitable for the in-line nucleophilic attack to the anomeric carbon. These observations might suggest that LNT was bound in a catalytically incompetent mode in the structure. The side chain of Glu-366 changes its conformation upon the substrate binding and makes a hydrogen bond with the O6 atom of the Gal moiety. It is unclear whether the difference between the Gal molecules in the two crystallographic structures is caused by an artifact during crystallization or reflects dynamics that could occur during catalysis. Nonetheless, it is noteworthy that the Gal moiety in the E318S-LNT structure adopts a slightly distorted ^O^*H*_5_-like (*ψ* ~ 324°) conformation. In the case of GH2 LacZ β-galactosidase from *Escherichia coli*, the Gal moiety of allolactose bound to subsite -1 took the ^3^*H*_4_ (*ψ* ~ 210°) conformation^[49]^. The β-galactosidic bond of allolactose in LacZ was in a pseudo-axial orientation that is suitable for the nucleophilic attack from the catalytic Glu residue, and a conformation itinerary through ^4^*C*_1_-^3^*H*_4_-^4^*C*_1_ was proposed for the catalysis. Since it has been suggested that sugar distortion is generally involved in the catalysis of β-glycosidases^[50]^, our observation of the sugar distortion in *Bi*Bga42A might suggest that a similar conformational change occurs during catalysis in GH42.

The leaving group trisaccharide moiety [lacto-*N*-triose II (LNT2), GlcNAcβ1-3Galβ1-4Glc] of LNT is recognized by the protein via both stacking and hydrogen bonding interactions. The sugar ring of the GlcNAc in subsite +1 forms a stacking interaction with the side chain of Trp-326 [Figure 2B]. The nitrogen atom of the *N*-acetyl group is recognized by a direct hydrogen bond with the carboxyl group of Glu-160 (the catalytic acid/base). The *N*-acetyl group is fixed by a hydrophobic patch created by the side chain of Met-262 and by an intramolecular hydrogen bond with the O2 atom of the Gal moiety of the Lac unit. The C6 hydroxymethyl group of the GlcNAc moiety, which faces solvent, is constrained in a *gg* conformation by a packing interaction with Phe-221 from the neighboring molecule of the trimer. Phe-221 is located on the tip of a long helix next to Trp-200 [Figure 1]. As for the Gal moiety of the Lac unit, no specific interaction with the protein was observed [Figure 2C]. In addition, the reducing-end Glc is recognized by two direct and one water-mediated hydrogen bonds. The direct contacts are formed between the O6 atom of the Glc moiety and the side chain of Arg-327 and between the O2 atom of the Glc moiety and the carbonyl oxygen of the peptide bond of Gly-266-Cys-267. Water-mediated hydrogen bonds from the O3 atom of the Glc moiety are formed with the nitrogen atom of the peptide bond of Cys-267-Thr-268. The Gal and Glc moieties of the Lac unit of LNT show higher B-factors than the Gal and GlcNAc moieties of the LNB unit of LNT, suggesting that the reducing-end Lac unit is relatively flexible [Supplementary Table 1]. Overall, the LNT molecule bound to the catalytic pocket of the E318S mutant is recognized by an extensive hydrogen bond network and several hydrophobic interactions which involve a residue from the neighboring subunit.

### Mutational analysis

We then introduced amino acid replacements into several positions described above to evaluate their importance in the reaction process. The substrates used were *p*NP-Gal, LNB, LNT, and LNnT. The values obtained for the WT enzyme were comparable with those reported in our previous study^[11]^.

*Subsite -1*: Alanine substitution for Arg-121 (R121A) increased the *K*_m_ values for the four substrates by 2- to 3-fold, while it reduced the *k*_cat_ values by 20- to 30-fold [Table 1]. The results suggest that Arg-121 contributes more to catalysis than to substrate binding. The Y287F substitution, which presumably causes the loss of a hydrogen bond with the Gal moiety of LNT, did not cause a marked alteration in the *K*_m_ values but caused a marked reduction in the *k*_cat_ values by 80- to 170-fold. The effect on the *k*_cat_ values for *p*NP-Gal was the third largest, following the nucleophile mutation (E318A) and the acid/base catalyst mutation (E160A), indicating that this residue plays a crucial role in the catalysis.

*Subsite +1*: Alanine substitution for Phe-221 (F221A), the residue from the neighboring subunit, slightly increased the *K*_m_ values for all substrates within a range less than 3-fold and decreased the *k*_cat_ values by 2- to 20-fold. The considerable effect was observed for LNB followed by LNT regarding the turnover, thus demonstrating the involvement of this residue in fixation of the GlcNAc moiety during catalysis. The M262G replacement had no remarkable effects on the substrate binding by the enzyme, but considerably affected the *k*_cat_ values, which decreased between 30- and 100-fold. The results suggest that, in addition to its importance in the hydrophobic patch formation near subsite +1, Met-262 plays an important role in maintaining the side chain orientation of Glu-160 (the acid/base residue) properly during catalysis, as suggested by the crystal structure. The replacement of Trp-326 with alanine had various effects on activity. While it increased the *K*_m_ value by 20-fold and decreased the *k*_cat_ value by 13-fold for *p*NP-Gal, it increased the *K*_m_ values by 1.3- to 4-fold and decreased the *k*_cat_ value by 100- to 600-fold for natural substrates. The greater extent of change in the *k*_cat_ values for natural substrates than for *p*NP-Gal indicates the importance of the sugar-protein stacking interaction at subsite +1 in the transition state. The W326A mutation affected the activity of LNB and LNT to a similar extent.

*Subsite +3*: When Arg-327 was replaced with alanine (R327A), a slight increase in the *K*_m_ values for LNT and LNnT was observed without considerably affecting the parameters for the other substrates. Thus, we predict that the interaction between the reducing-end Glc moiety and Arg-327 may also occur between the protein and LNnT.

### Conservation of the amino acid residues involved in the substrate recognition within structure-determined GH42 β-galactosidases and within *Bi*Bga42A homologs from several *Bifidobacterium* species

**Figure 4:**
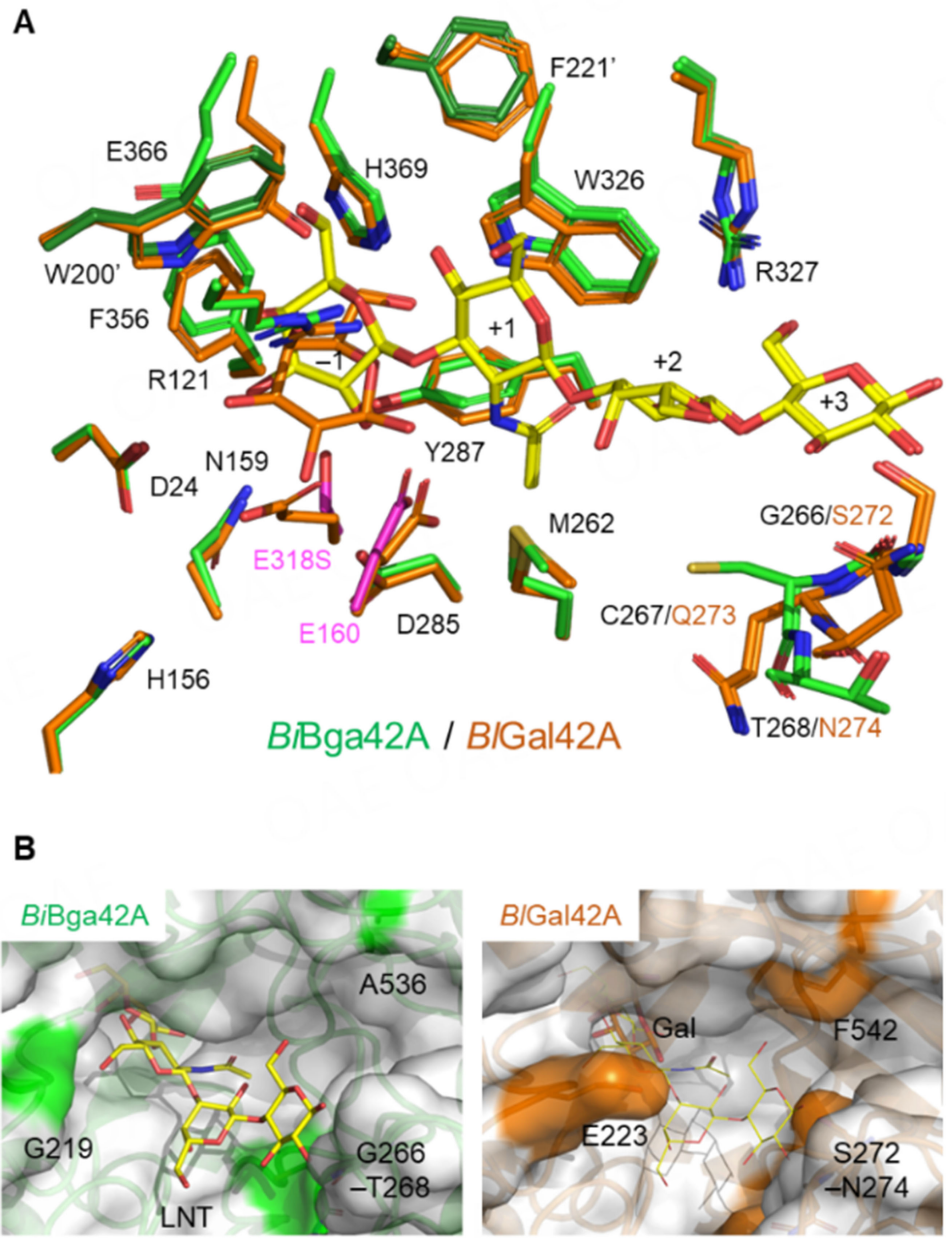
Comparison of the active site structures between *Bi*Bga42A and *Bl*Gal42A. (A) Superimposition of the active sites of *Bi*Bga42A (E318S-LNT, green for protein and yellow for LNT) and *Bl*Gal42A (orange for protein and Gal); (B) Molecular surface of *Bi*Bga42A (left) and *Bl*Gal42A (right). *Right*, LNT in *Bi*Bga42A is superimposed as thin sticks. Surface areas of the residues contributing to shaping the different entrance sizes are colored green or orange.*Bi*Bga42A: a glycoside hydrolase family 42 β-galactosidase; LNT: lacto-*N*-tetraose; *Bl*Gal42A: β-1,6-1,3-galactosidase.

To date, crystal structures of GH42 members are reported in the apo- and/or Gal-complexed forms only. The E318S-LNT thus represents the first GH42 structure in complex with a substrate. The subsequent mutational analysis also identified several amino acid residues important for substrate recognition and catalysis. We chose all of the above-mentioned amino acid residues and examined their conservation within the GH42 β-galactosidase members whose crystal structures have been determined^[23-30]^. Note that the amino acid residues that interact with the sugar (LNT) through the peptide backbone (Gly-266-Thr-268) were not considered in the conservation analysis. It should also be mentioned that structural signatures that discriminate between β-galactosidases and α-arabinopyranosidases within GH42 members have been reported previously^[31]^. Figure 3A shows the conservation pattern of the selected amino acid residues. Among the 16 amino acid residues, including the two catalytic residues (E160 and E318), 12 residues were identical among the members. By reflecting distance in the phylogenetic tree [Figure 3B], Bga from *Halorubrum lacusprofundii*^[29]^, β-Gal-ase from *Marinomonus* sp.^[30]^, and A4-β-Gal from *T. thermophilus*^[23]^ have different amino acid residues at 4 positions (corresponding to residues 24, 156, 221, and 327 of *Bi*Bga42A). Those residues are conserved among the three enzymes except for residue 327, which interacts with the distal reducing-end Glc moiety of LNT in *Bi*Bga42A. Unfortunately, as substrate specificities of these three enzymes have not been reported, we are unable to discuss how these amino acid replacements are linked with specificity differences among the structure-determined GH42 β-galactosidases. However, we were aware that there is a striking difference in the substrate specificity between *Bi*Bga42A and *Bl*Gal42A, which have identical amino acid residues at the selected 16 positions [Figures 3A and 4A] and share 62% overall amino acid sequence identity. Both enzymes efficiently hydrolyze β-(1→6)- and β-(1→3)-linkages and, to a moderate extent, β-(1→4)-linkages when disaccharides were used as substrates^[12,25]^. However, while *Bi*Bga42A can accept tetrasaccharides, *Bl*Gal42A shows very limited activity towards tetrasaccharides. For example, the catalytic efficiencies (*k*_cat_/*K*_m_) of *Bi*Bga42A towards 3-galactobiose (Galβ1-3Gal) and 3-galactobiosyllactose (Galβ1-3Galβ1-3Galβ1-4Glc) were 422 and 842 mM^-1^s^-1^, respectively^[12]^. In contrast, while *Bl*Gal42A hydrolases 3-galactobiose with the catalytic efficiency of 52 mM^-1^·s^-1^, it was inactive on 3-galactobiosyllactose^[25]^. Moreover, in contrast to *Bi*Bga42A, which efficiently hydrolyzed LNT, *Bl*Gal42A was incapable of hydrolyzing LNT^[12]^. When the active site structures of *Bi*Bga42A and *Bl*Gal42A were superimposed, side chains of the conserved 16 residues were found to have almost the same conformation except for Glu-366, which is displaced upon LNT binding [Figure 4A]. However, the main chain trace and the side chain conformation of Ser-272-Asn-274 in *Bl*Gal42A are different from those of Gly266-Thr268 in *Bi*Bga42A. Upon examination of the surface models of the two structures, we found that the catalytic pocket of *Bl*Gal42A is narrower than that of *Bi*Bga42A [Figure 4B]. The side chain of Ser-272 makes the pocket of *Bl*Gal42A slightly tighter. The protruding side chain of Glu-223 located on a loop of *Bl*Gal42A especially occludes the entrance of the pocket. Phe-542, which is located in the loop extending from the other side, also covers the entrance of the catalytic pocket of *Bl*Gal42A. *Bi*Bga42A substitutes Gly-219 and Ala-536 for Glu-223 and Phe-542 of *Bl*Gal42A, respectively. These bulky residues might interfere with the efficient access of longer oligosaccharide substrates to the catalytic site of *Bl*Gal42A.

We also examined the conservation of these amino acid residues among the *Bi*Bga42A homologs of several *Bifidobacterium* species that reside in the gastrointestinal tract (GIT) of humans and non-human mammals [Table 2]. Genome sequence-completed, type strains of 14 species/subsp. were selected for the analysis. Twelve species/subsp. were found to possess *Bi*Bga42A homologs with 62%-96% identity. The lowest hit (62%) was observed for *Bl*Gal42A. Overall identities among the retrieved 12 homologs were between 59% and 96%, and all 16 residues involved in substrate recognition [Figure 3A] were conserved, as revealed by ClustalW analysis^[44]^. Interestingly, the above-mentioned Gly-219 is also conserved among the homologs including *Bl*Gal42A in the sequence alignment [Figure 4B and Supplementary Figure 3]. Notably, *Bl*42GalA has one amino acid insertion in the corresponding loop, which might result in the protrusion of Glu-223 at the entrance. The twelve homologs possess a glutamic acid or aspartic acid residue in the corresponding position in the sequences. The multiple alignments also revealed sequence variance at the Gly266-Thr268 and Ala-536 positions among the homologs. Further enzymatic characterization and mutational analysis are necessary to examine how these homologs efficiently hydrolyze LNT and related tetra- or longer oligosaccharides.

**Table 2:** Conservation of *Bi*Bga42A homologs among several *Bifidobacterium* species

**Species (Genome sequence-** **completed type strain)**	**Locus tag**	**Identity (%)**
*B. adolescentis* ATCC 15703	BAD_1603	515/690 (75%)
*B. angulatum* JCM 7096	BBAG_0066	527/691 (76%)
*B. animalis* subsp. *animalis* ATCC 25527	nr^a^	
*B. animalis* subsp. *lactis* DSM 10140	BALAT_0484 (*Bl*Gal42A)	430/692 (62%)
*B. bifidum* JCM 1255	BBBF_1344	521/690 (76%)
*B. breve* JCM 1192	BBBR_0453	660/691 (96%)
*B. catenulatum* subsp. *catenulatum* JCM 1194	BBCT_0461	522/690 (76%)
*B. catenulatum* subsp. *kashiwanohense* JCM 15439	BBKW_0505	523/690 (76%)
*B. dentium* JCM 1195	BBDE_0630	517/690 (75%)
*B. eulemuris* DSM 100216	BE0216_04680	569/689 (83%)
*B. lemurum* DSM 28807	BL8807_06925	565/690 (82%)
*B. longum* subsp. *longum* JCM 1217	BLLJ_0443	661/691 (96%)
*B. pseudocatenulatum* JCM 1200	BBPC_0515	517/690 (75%)
*B. pseudolongum* subsp. *globosum* DSM 20092	nr	

^a^None retrieved. *Bi*Bga42A: a glycoside hydrolase family 42 β-galactosidase.

### Concluding remarks

GH42 enzymes, which are found exclusively in microbes, i.e., bacteria, archaea, and fungi, were previously thought to be involved in the degradation of plant-derived galactooligosaccharides released from galactans^[51,52]^. However, a study on *Bi*Bga42A, which was first described to be specific for LNT^[11]^ but later found to act on a variety of both β-(1→3)- and β-(1→6)-linked galactosides^[12]^, revealed that GH42 β-galactosidases target oligosaccharides of both plant and animal origins. A recent *in silico* analysis also demonstrated that the expression of most GH42 β-galactosidase genes is not subject to the regulation by NagR, a global transcriptional regulator of animal host-glycan degradation genes, in the genus *Bifidobacterium*^[53]^. In *B. infantis*, the transcriptional levels of the three GH42 genes were not affected by the carbon sources used for cultivation, which may represent “enzymatic preparedness in gut microbes” as mentioned previously^[54]^.

Using *Bi*Bga42A, a well-characterized GH42 member, our study presents, for the first time, the structure of a GH42 enzyme complexed with its substrate. Comparison between the two liganded structures showed that the Gal moiety of LNT occupies the subsite -1 that is sterically different from that observed for the Gal in E160A/E318A-Gal and takes a different sugar conformation. The result may represent the dynamics in the catalytic cycle adopted by GH42 enzymes and may explain the lack of transglycosylation activity of anomer-retaining GH42 members. The donor sugar (Gal) bound at subsite -1 and an acceptor sugar bound at + subsite(s) may not be arranged suitably for transglycosylation to proceed. Overall, our study warrants further structural analysis of GH42 members with varied substrates and inhibitors to understand the catalytic mechanism and the molecular basis of different substrate specificities among the members, which may help us design novel prebiotics with distinct sugar linkages and compositions.

## DECLARATIONS

### Availability of data and materials

Atomic coordinates and structure factors of WT-GOL, E160A/E318A-Gal, and E318S-LNT of the Bga42A protein from *B. infantis* ATCC15697 have been deposited in the PDB under accession numbers 8IBR, 8IBS, and 8IBT, respectively. All materials except for LNT and LNnT, which are gifts from Glycom A/S, are available upon reasonable request.

### Copyright

© The Author(s) 2023.
