# Supplementary material for: Substrate recognition mode of a glycoside hydrolase family 42 β-galactosidase from Bifidobacterium longum subspecies infantis (BiBga42A) revealed by crystallographic and mutational analyses
Source: Microbiome Res Rep. 2023 May 26;2(3):20. doi: 10.20517/mrr.2023.14 (PMC10688820; doi:10.20517/mrr.2023.14)
Supplement: Supplementary file 1 [file mrr-2-3-20-SupplementaryMaterials.pdf]

Supplementary Table 1. Crystallographic data statistics of *Bi*Bga42A

Data set	WT + GOL	E160A/E318A + Gal	E318S + LNT
Data collection [a]			
Space group	*P* 3 2 1	*C* 2	*P* 3 2 1
Unit cell (Å, °)	$a = b = 145.642$, $c = 66.845$	$a = 234.470$, $b = 182.691$, $c = 143.263$, $\beta = 125.730$	$a = b = 166.432$, $c = 149.459$
Resolution (Å)	47.67–1.70 (1.73–1.70)	48.57–1.90 (1.93–1.90)	48.04–2.20 (2.24–2.20)
Total reflections	785,834 (5,749)	1,409,212 (44,332)	909,431 (44,028)
Unique reflections	85,479 (2,985)	376,638 (15,738)	121,062 (5,977)
R_{merge}	0.086 (0.586)	0.097 (0.935)	0.134 (0.916)
R_{pim}	0.029 (0.464)	0.076 (0.667)	0.052 (0.362)
Mean $I/\sigma(I)$	16.3 (1.4)	9.5 (1.1)	12.8 (2.5)
$CC_{1/2}$	0.998 (0.784)	0.997 (0.545)	0.996 (0.633)
Completeness (%)	95.5 (63.5)	98.3 (83.0)	100.0 (99.9)
Multiplicity	9.2 (1.9)	3.7 (2.8)	7.5 (7.4)
Mol/ASU [b]	1	6	2
Refinement			
Resolution (Å)	45.91-1.70	48.61–1.90	48.05–2.20
No. of reflections	85,468	376,630	121,060
R_{work}/R_{free} [c]	0.161/0.186	0.187/0.228	0.146/0.175
Twin fraction	–		0.347
Number of atoms			
Amino acids	5,477	32,789	10,967
Ligands	13 (glycerol + PEG)	72 (α-Gal x 6)	96 (LNT x 2)
Waters	670	2,870	650
B-factors (Å^2)			
Amino acids	15.04	26.14	30.08
Ligands	21.68	24.78	45.08 (33.5, 39.9, 52.6, 50.1; 38.1, 39.9, 56.1, 52.1) [d]
Waters	26.02	29.58	32.54
RMSD from ideal values			
Bond lengths (Å)	0.0115	0.0093	0.0125
Bond angles (°)	1.78	2.22	1.86
Ramachandran plot (%)			
Favored	96.37	96.71	96.31
Allowed	3.05	2.73	2.90
Outlier	0.58	0.56	0.80
PDB code	8IBR	8IBS	8IBT

[a]Values in parentheses are for the highest resolution shell.

[b]Number of molecules per asymmetric unit.

[c]R_{free} was calculated for a randomly chosen 5% of reflections, which were not used for structure refinement, and R_{work} is calculated for the remaining reflections.

[d]Average B-factor (Å^2) of each sugar moiety of the two LNT molecules in the asymmetric unit. Values for Gal, GlcNAc, Gal, and Glc in chains A and B are shown.

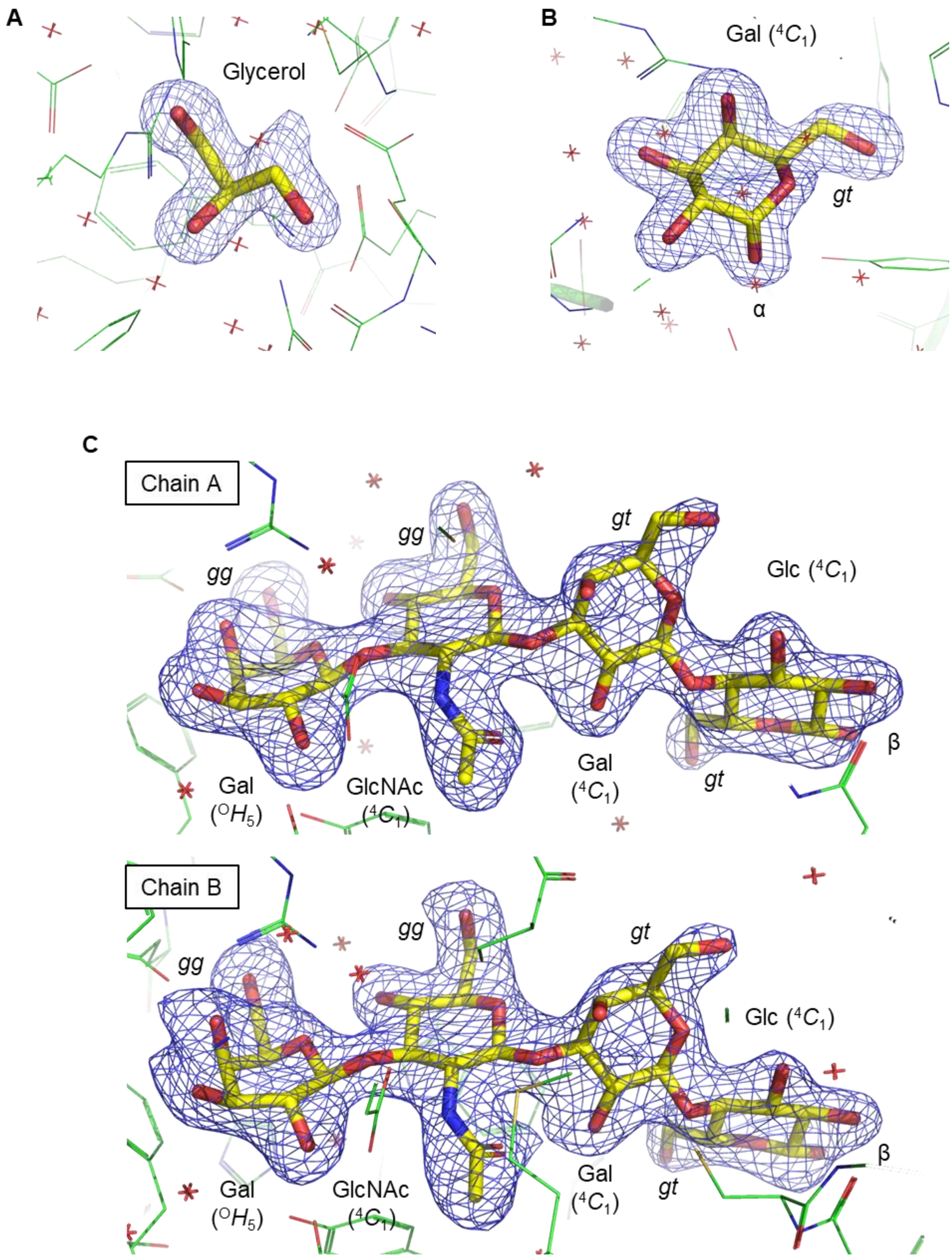

Supplementary Figure 1. Electron density maps in the active site. Polder maps are shown. **(A)** Glycerol bound to subsite –1 of WT-GOL (5σ). **(B)** Gal bound to subsite –1 in chain A of E160/E318A-Gal (4σ). **(C)** LNT bound to subsites from –1 to +3 in chains A (upper) and B (lower) of E318S-LNT (3σ). Anomer configuration and conformation of the sugars are indicated. *gg* and *gt*: Rotamer conformation of the C6 hydroxymethyl group.

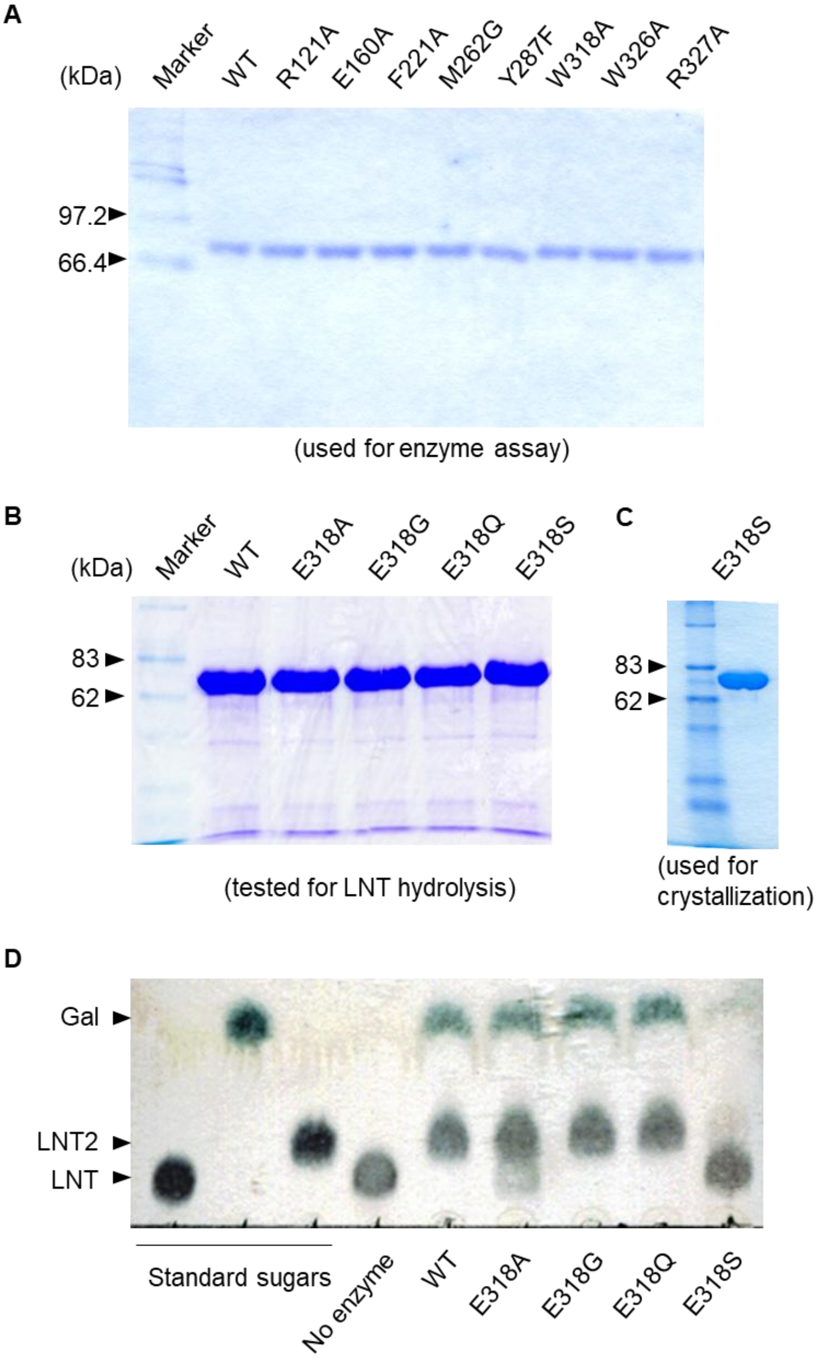

Supplementary Figure 2. *Bi*Bga42A variants prepared for enzyme assay and crystallization. (A–C) The results of sodium dodecyl sulfate-polyacrylamide gel electrophoreses of *Bi*Bga42A variants prepared for enzyme assay **(A)**, examining LNT-hydrolyzing activity **(B)**, and crystallization **(C)**. The gels were stained with Coomassie Brilliant Blue R-250. **(D)** LNT-hydrolyzing activities of *Bi*Bga42A E318A, G, Q, and S mutants. The enzyme preparation shown in **(B)** was used for the analysis. The reaction mixture consisting of 100 mM MOPS (pH6.5), 0.05% Tween-20, 5 mM LNT, and 9 mg/mL each enzyme was incubated at 30ºC for 12 h. The products were analyzed by a thin-layer chromatography. LNT2: lacto-*N*-triose II (GlcNAcβ1-3Galβ1-4Glc).

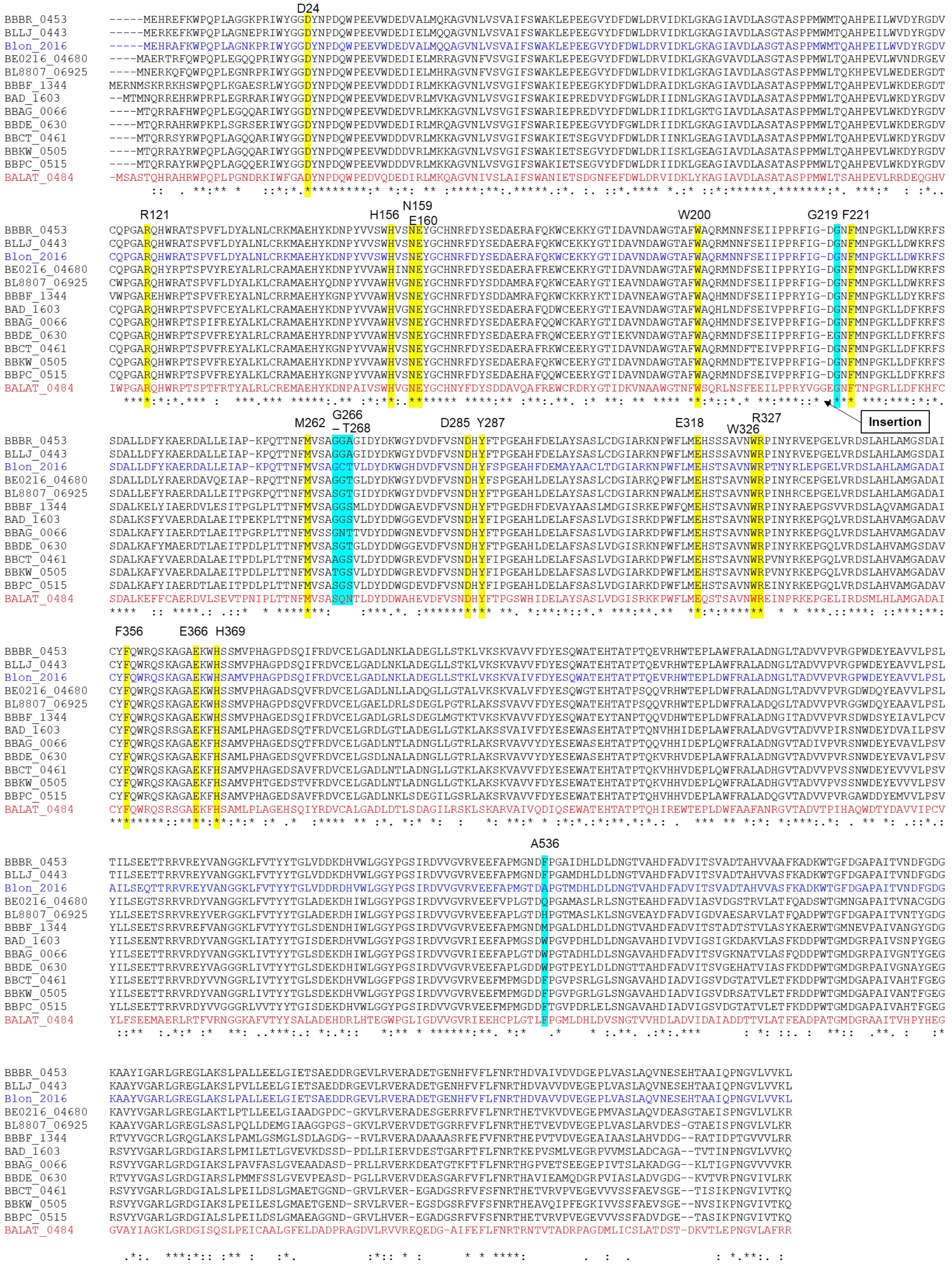

Supplementary Figure 3. Sequence alignment of *Bi*Bga42A homologs from several *Bifidobacterium* species. Twelve homologs were aligned using ClustalW (see Table 2). Overall identities are 62–96% among the homologs. The residues involved in LNT recognition and hydrolysis in *Bi*Bga42A are highlighted in yellow, while the residues shaping the entrance of the catalytic pocket are highlighted in cyan. One amino acid insertion in the loop of *Bl*Gal42A is indicated by an arrow. The residue numbers are based on *Bi*Bga42A. *Bi*Bga42A and *Bl*Gal42A are shown in blue and red, respectively.
